# Novel Therapeutic Strategies for Reducing Right Heart Failure Associated Mortality in Fibrotic Lung Diseases

**DOI:** 10.1155/2015/929170

**Published:** 2015-10-25

**Authors:** Ayodeji Adegunsoye, Matthew Levy, Olusegun Oyenuga

**Affiliations:** ^1^Section of Pulmonary & Critical Care Medicine, Department of Medicine, University of Chicago, Chicago, IL 60637, USA; ^2^Department of Cardiology, Deborah Heart and Lung Center, Browns Mills, NJ 08015, USA; ^3^Section of Cardiology, Department of Medicine, University of Chicago, Chicago, IL 60637, USA

## Abstract

Fibrotic lung diseases carry a significant mortality burden worldwide. A large proportion of these deaths are due to right heart failure and pulmonary hypertension. Underlying contributory factors which appear to play a role in the mechanism of progression of right heart dysfunction include chronic hypoxia, defective calcium handling, hyperaldosteronism, pulmonary vascular alterations, cyclic strain of pressure and volume changes, elevation of circulating TGF-*β*, and elevated systemic NO levels. Specific therapies targeting pulmonary hypertension include calcium channel blockers, endothelin (ET-1) receptor antagonists, prostacyclin analogs, phosphodiesterase type 5 (PDE5) inhibitors, and rho-kinase (ROCK) inhibitors. Newer antifibrotic and anti-inflammatory agents may exert beneficial effects on heart failure in idiopathic pulmonary fibrosis. Furthermore, right ventricle-targeted therapies, aimed at mitigating the effects of functional right ventricular failure, include *β*-adrenoceptor (*β*-AR) blockers, angiotensin-converting enzyme (ACE) inhibitors, antioxidants, modulators of metabolism, and 5-hydroxytryptamine-2B (5-HT2B) receptor antagonists. Newer nonpharmacologic modalities for right ventricular support are increasingly being implemented. Early, effective, and individualized therapy may prevent overt right heart failure in fibrotic lung disease leading to improved outcomes and quality of life.

## 1. Introduction

The interstitial lung diseases (ILD) comprise a heterogeneous group of pulmonary disorders with similar clinical and radiographic characteristics. Etiologies range from identifiable environmental and medication exposures to connective tissue diseases. A significant portion of ILD remains idiopathic amongst which the progressive fibrotic lung diseases are the most clinically challenging and carry significant mortality burden [1]. This category includes idiopathic pulmonary fibrosis (IPF), fibrotic nonspecific interstitial pneumonia (FNSIP), chronic hypersensitivity pneumonitis (CHP), and connective tissue disease related ILD (CTD-ILD).

Treatment of ILD is usually targeted at avoiding potential etiologic factors, correction of hypoxemia, and blunting the inflammatory response that ultimately results in fibrosis. Despite advances in medicine, the incidence and mortality of IPF, one of the more common fibrotic lung diseases, continue to rise worldwide. Cardiovascular comorbidities like right heart failure (RHF) and pulmonary hypertension (PH) account for a large proportion of these deaths [2] and an effective approach to the management of these comorbid conditions constitutes an appealing target for improving outcomes and quality of life in this group of patients.

## 2. Pulmonary Hypertension and Right Ventricular Dysfunction in Fibrotic Lung Diseases

The development of PH in IPF patients has been associated with several mechanistic factors such as poor resting gas exchange, low diffusing capacity of the lungs for carbon monoxide (DLCO), increased desaturation with exercise, and cardiovascular mediated exercise limitation [3–5]. Although the gold standard for the diagnosis of PH is right heart catheterization, PH can also be assessed with noninvasive modalities with prognostic implications.

More than 60% of patients with end-stage IPF demonstrate mean pulmonary artery pressure (mPAP) >25 mmHg [5–7]. Though the mPAP exceeds 40 mmHg in a fraction of these patients (~9%) [8], the extent of lung function impairment has not been shown to correlate significantly with severity of PH [8]. PH may rapidly progress in the later stages of IPF and other fibrotic lung diseases [4, 6]. The prognostic implications of PH in fibrotic lung disease have been demonstrated with mPAP, pulmonary vascular resistance (PVR), and cardiac index (CI). CI below 2.4 L/min/m^2^ has been correlated with a limited life expectancy of several months [9–11]. Radiographic demonstration of right heart dilation and elevated serum levels of brain natriuretic peptide (BNP) also have prognostic significance with worsening PH in these patients [3–5]. Transthoracic echocardiogram (TTE) therefore remains a useful tool in the evaluation of PH and is currently the recommended method for early detection [12, 13]. Systolic PAP measurements by TTE are sensitive (79–100%) and specific (60–98%) for detection of PH especially in the presence of tricuspid regurgitation [14, 15]. Patients with chronic fibrotic lung disease may however experience wide variations in TTE estimations of sPAP necessitating the implementation of more accurate modalities in identification of patients at risk [16, 17]. The current guidelines for echocardiographic assessment of the right heart in adults also recommend the use of tricuspid annular plane systolic excursion (TAPSE) (reference range 1.5–2.0 cm) also referred to as tricuspid annular motion (TAM), a simple and easily reproducible technique which provides measurements of right ventricular annular systolic excursion in a longitudinal plane when evaluated in a standard apical 4-chamber view [18]. Stress echocardiography and newer techniques such as right ventricular function parameters as measured by tissue Doppler (e.g., RV E/Em index) or right ventricular isovolumic relaxation time (RV-IVRT) may yield better indices and improved correlation with survival [10, 19, 20]. The combination of more than one measure of right ventricular function may provide more reliable indices to detect abnormal function [18].

Cardiac MR is an increasingly attractive modality for assessing the pulmonary artery and right ventricle in patients with pulmonary fibrosis [21, 22]. Contrast-enhanced CT scans can also be used to assess right ventricular size; interventricular septal deviation and demonstration of contrast reflux into the inferior vena cava in individuals with PH are specific for the presence of tricuspid regurgitation [23].

## 3. Etiologic Factors in the Mechanism of Onset and Progression of Right Ventricular Dysfunction

Right ventricular failure commonly complicates chronic PH and is the strongest prognostic factor in this group of patients [24]. Right ventricular failure typically follows RV-PA uncoupling, which occurs when the elevated pulmonary vascular resistance exceeds the intrinsic contractility of the right ventricle. Unlike pulmonary arterial hypertension (PAH) in which disease severity of the distal pulmonary vasculature is thought to play key roles in occurrence of right ventricular hypertrophy and failure, the underlying mechanisms of right ventricular dysfunction in fibrotic lung diseases are not fully understood [25].

**Figure 1:**
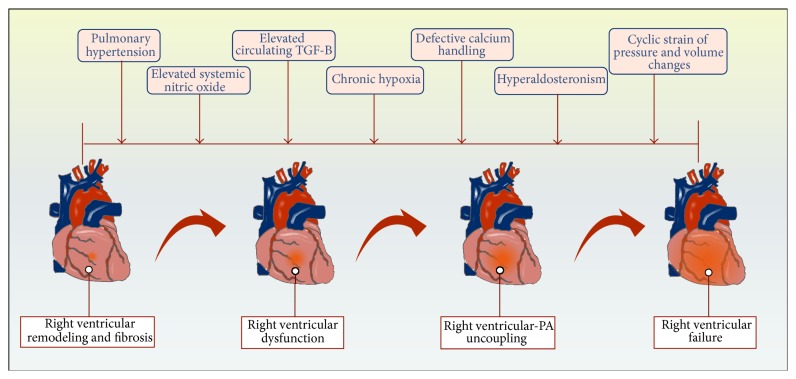
Factors associated with progression to right ventricular failure in fibrotic lung disease.

Experimental animal models of chronic PH have demonstrated the presence of diastolic dysfunction as an early marker for right ventricular remodeling and increased right ventricular fibrosis even in the absence of heart failure. Defective calcium handling, hyperaldosteronism, and RV-PA uncoupling herald the onset of overt right ventricular failure [26] (Figure 1). Other studies demonstrate that absence of caveolin-1, a structural protein predominantly expressed in fibroblast and endothelial cells, results in marked secondary right ventricular hypertrophy following significant pulmonary hypertension, with elevation of systemic NO levels [27]. This elevation in systemic NO levels which characterizes cardiomyopathy and PH in human and animal models may reduce myocardial contractility and mediate the deleterious effects of various cytokines on intrinsic myocardial inotropic activity [28, 29] (Figure 1).

Significantly elevated circulating levels of TGF-*β*, a profibrotic mediator that promotes aberrant gene expression and abnormal collagen deposition such as that occurring in pulmonary fibrosis, have been demonstrated in patients with dilated cardiomyopathy and worsening congestive heart failure [30]. TGF-*β* and IL-10 were also associated with increased pulmonary microvascular pressure and are thought to play key roles in cardiac and pulmonary fibrotic remodeling [30, 31]. TGF-*β* produced by cardiomyoblasts have been demonstrated to induce airway squamous metaplasia through Smad signaling, a mechanism that could worsen airflow obstruction in individuals with heart failure [32].

Chronic hypoxia appears to modify the response of the right ventricular to pressure overload by the uncoupling of endothelial nitric oxide synthase thereby resulting in an accelerated decline in right ventricular function [33] (Figure 1). Cyclic strain of pressure and volume changes on the right heart result in increased right ventricular wall tension promoting development of myocardial hypertrophy [34–36]. The increased stretch of the ventricular wall upregulates the transcription of the BNP gene thereby increasing cardiomyocyte secretion of BNP [37]. Its inactive metabolite NT-pro-BNP correlates with measures of right ventricular dysfunction as determined by CMR or echocardiography and elevated baseline values (>1,685 pg·mL^−1^) predict poor prognosis [38–40].

Multiorgan fibrotic infiltration has also been described to result in right ventricular dysfunction. Alstrom syndrome, an autosomal recessive condition characterized by blindness, dilated cardiomyopathy, and metabolic abnormalities, is associated with fibrotic lung disease, glomerulofibrosis, and sensorineural hearing loss [41]. Myocardial evaluation of these patients with cardiac magnetic resonance imaging displays an absence of fluid or fatty infiltration. Instead all patients demonstrate a patchy distribution of myocardial fibrosis involving the left and right ventricles and concomitant impairment of biventricular function [41, 42].

## 4. Mortality from Right Heart Failure in Fibrotic Lung Disease

The interdependent physiologic mechanisms linking right heart failure to fibrotic lung disease reflect the anatomic proximity of these organs and the overall contribution to morbidity and mortality in patients with both conditions. As the worldwide aging population increased over the last few decades, hospitalizations for cardiovascular disease have also risen, a significant proportion of these due to heart failure [43–45]. The worldwide increase in the prevalence of heart failure and the 5-year mortality carried by this diagnosis exerts considerable socioeconomic impact on the affected individuals and the overall health care system [46]. Similarly, the occurrence of fibrotic lung disease may severely limit the life expectancy of affected patients such as the case in individuals with idiopathic pulmonary fibrosis where the median survival is 2-3 years rivaling that of several cancers [47]. A significant fraction of deaths in this subset of patients has been attributed to heart failure [48].

The contribution of right heart failure to mortality in fibrotic lung diseases involves a broad interplay of several pathophysiologic mechanisms such as structural alteration in the pulmonary vasculature with hemodynamic consequences, disequilibrium of pulmonary fluid homeostasis, occurrence of sleep disordered breathing, and distortion of pulmonary mechanics as evident on lung function testing.

### 4.1. Pulmonary Vascular Alterations

Despite high pulmonary pressures, which characterize right heart failure in fibrotic lung disease, these patients are less prone to developing pulmonary edema. Studies from autopsy findings and biopsy specimens suggest that the capillary bed undergoes several alterations including increased capillary dilation and thickness of the basement membrane, thickening of the tunica intima, and muscularization and circumferential fibrosis of the pulmonary vessels. These changes are accompanied by increased alveolar wall thickening following excessive collagen deposition, adjacent airway compression, and bronchial smooth muscle hypertrophy, processes amplified in the presence of underlying fibrotic lung disease [49–51]. These vascular alterations appear to decrease capillary filtration rate and increase the level at which hydrostatic pressure produces pulmonary edema [49, 51].

### 4.2. Impairment of Pulmonary Fluid Homeostasis and Acute Pulmonary Edema

Progressive left heart failure increases left atrial pressure transmitted via pulmonary veins and capillaries to the right heart manifesting as pulmonary hypertension and ultimately right heart failure. Long standing pulmonary hypertension increases tolerance of high pressures with a lower tendency to develop pulmonary edema. However a rapid rise in the capillary wedge pressure may result in pulmonary edema even at low pressures. Elevated hydrostatic forces may partially disrupt the alveolar-capillary unit resulting in pulmonary capillary stress fracture and eventual pulmonary edema [49, 52, 53].

### 4.3. Sleep Disordered Breathing

The presence of sleep disordered breathing commonly complicates heart failure and the associated sympathetic overactivity results in functional impairment and increased mortality [54, 55]. Up to a third of patients with advanced heart failure exhibit central sleep apnea with increased morbidity and mortality [56, 57]. Also, the presence of obstructive sleep apnea is an independent risk factor for developing pulmonary hypertension and eventual cor pulmonale [36]. The importance of recognition of sleep-related breathing disorders in idiopathic pulmonary fibrosis has resulted in its categorization by the International Classification of Sleep Disorders (ICSD) to a unique group, “sleep disorders with sleep-related hypoventilation and hypoxemia in parenchymal or vascular lung diseases” [58] most recently reclassified in 2014 to the specific subgroup, “sleep-related hypoxemia disorder” [59]. This is a result of the peculiar pattern of oxygen desaturation that characterizes this group of patients. These individuals exhibit multiple phasic oxygen desaturations which occur frequently from hypoventilation and may eventually lead to sleep fragmentation and poor quality of sleep [60].

### 4.4. Impact of Heart Failure on Pulmonary Function Testing

Studies examining the altered lung function in patients with decompensated heart failure are few. Patients acutely hospitalized for heart failure appear to have increased pulmonary resistance and demonstrate reduction in lung compliance, total lung capacity, FEV1, and FVC with no change in DLCO when compared to subsequent follow-up testing [61]. FEV1 and FVC have been demonstrated to be independent predictors of mortality in this cohort [62, 63]. The restrictive physiology of pulmonary fibrosis may exacerbate these observed changes in the presence of concomitant heart failure. The contribution of heart failure to restrictive lung disease may be explained by the increased heart size in a fixed thoracic cavity thus reducing the functional lung volumes [64].

## 5. Heart Failure Exacerbating Fibrotic Interstitial Lung Diseases

Patients with fibrotic interstitial lung diseases often undergo acute respiratory decline, which may be due to the presence of congestive heart failure, venous thromboembolic disease, or infectious etiologies [65]. When careful exclusion of these causes has been performed, the acute respiratory deterioration is attributed to unexplained causes and is then termed acute exacerbation of interstitial lung disease (AE-ILD) such as acute exacerbations of idiopathic pulmonary fibrosis (AE-IPF) [66–71]. Because heart failure commonly complicates the clinical course of fibrotic lung disease, patients who present with rapidly worsening pulmonary symptoms, oxygen desaturation, and acute onset of radiographic infiltrates in the past month should undergo thorough detailed clinical and transthoracic echocardiographic assessment of ventricular function with exclusion of pulmonary hypertension and venous thromboembolic disease as part of their diagnostic workup [65, 72]. The therapeutic approach to management in these cases should target identifiable cardiac causes of respiratory decline in patients with fibrotic lung disease.

## 6. Treatment of Pulmonary Hypertension Associated with Right Ventricular Dysfunction

The sustained pulmonary vasoconstriction and progressive vascular remodeling that characterizes PH result in irreversible right heart dysfunction and ultimately acute decompensated right heart failure associated with high inhospital mortality [73–75]. The coexistence of chronic pulmonary disease and dysregulation of cellular proliferation may accelerate the World Health Organization (WHO) Functional Class (FC) decline of these patients into WHO-FC III or IV thus worsening survival outcomes [76–79].

The approach to treatment for these patients includes the use of oxygen and diuretics, as necessary, and anticoagulants in those individuals where specifically indicated [80, 81]. It should however be noted that the use of pulmonary vasodilators in lung fibrosis may contribute to worsening of gas exchange by inhibiting hypoxic pulmonary vasoconstriction [82].

### 6.1. Idiopathic Pulmonary Fibrosis and Pulmonary Hypertension

Multiple studies evaluating the utility of pulmonary vasoactive agents in patients with IPF and other fibrotic lung diseases have failed to demonstrate significant mortality benefits and in certain instances demonstrated harmful effects Table 2. This may be due to the absence of demonstrable vasoreactivity in PH-IPF patients thus limiting the utility of pulmonary vasodilators such as calcium channel blockers. Other limitations of these studies included a focus on short-term parameters, retrospective study design, and lack of randomization or inclusion of a placebo arm.

BUILD- (Bosentan Use in Interstitial Lung Disease-) 1 and BUILD-3 trials, which evaluated the effect of bosentan, a dual endothelin-1 receptor antagonist, in IPF failed to demonstrate a significant decrease in the time to IPF worsening [83, 84]. Macitentan, a novel dual endothelin receptor antagonist approved by the US FDA for treatment of PAH, was evaluated for the treatment of IPF in the MUSIC (Macitentan Use in an Idiopathic Pulmonary Fibrosis Clinical Study) trial [85]. Though this medication was well tolerated, the study revealed no significant difference in survival, lung function, or time to disease worsening.

In the ARTEMIS-IPF (Randomized, Placebo-Controlled Study to Evaluate Safety and Effectiveness of Ambrisentan in IPF) trial, subgroup analysis of patients treated with ambrisentan based on their PH status demonstrated no significant effect in those with mPAP >25 mmHg; rather they seemed to have disease progression and increased hospitalization for respiratory causes [86]. The more recent BPHIT (Bosentan in Pulmonary Hypertension Associated with Fibrotic Idiopathic Interstitial Pneumonia) trial which evaluated the safety and clinical efficacy of bosentan in patients with PH and fibrotic idiopathic interstitial pneumonia revealed no demonstrable difference in symptoms, functional capacity, or pulmonary hemodynamics over a 16-week period [87]. A subgroup analysis of patients enrolled in the STEP-IPF (Sildenafil Trial of Exercise Performance in Idiopathic Pulmonary Fibrosis) evaluated those patients with right ventricular systolic dysfunction but not right ventricular hypertrophy and found that those who received sildenafil demonstrated some improvement in 6-minute-walk distance but no difference in mortality or rate of acute exacerbations [88]. Further, a small pilot trial examining the effect of Riociguat on pulmonary hemodynamics in patients with PH and ILD of any cause demonstrated an acceptable safety profile [89].

Subsequently, the 2015 ATS/ERS/JRS/ALAT clinical practice guidelines strongly recommend that ambrisentan should not be used in patients with IPF regardless of the presence or absence of PH [90]. Also given the lack of mortality benefits and likelihood of net harm with the use of sildenafil, current recommendations are that sildenafil should not be used for treatment of IPF. The variability in reported outcomes across trials, increased cost, and imprecise estimates of their effect led to a recommendation against the use of bosentan or macitentan for the treatment of IPF. However, the guidelines note that these medications may benefit patients with PH-IPF more than other IPF patients.

While the previous 2011 ATS/ERS/JRS/ALAT clinical practice guidelines argued against treatment of PH in patients with IPF, the most recent update in 2015 makes no specific recommendation regarding this cohort and notes the lack of sufficient evidence to guide the clinical decision making process [90, 91]. Well-designed clinical trials of novel PH agents with acceptable safety profiles would help to determine the differential effect of treating PH in patients with IPF.

### 6.2. Pulmonary Hypertension and Other Fibrotic Lung Diseases

Fibrotic lung disease often results in PH (WHO Group 3), which may rapidly progress in the advanced stages [82]. However, some studies have shown that a reduction in cardiac index <2.4 L/min/m^2^ rather than mPAP predicts poor survival, thus indicating that coexisting ventricular dysfunction may be of prognostic significance [82]. The current guidelines recommend that, in addition to long-term oxygen therapy to keep arterial oxygen saturation above 90%, treatment of these patients should be focused on the underlying lung disease rather than the vascular component [82]. While it has been suggested that inhaled vasodilators may preferentially access those areas of the lungs with better ventilation and thereby improve oxygenation, supporting evidence in the form of large well-designed clinical trials is lacking.

Some patients with fibrotic lung disease may coincidentally develop PAH (WHO Group 1) as opposed to PH resulting from fibrotic lung disease (WHO Group 3) leading to uncertainty in patient classification [82, 92]. Occasionally patients with systemic sclerosis who develop pulmonary fibrosis and PH may demonstrate similar pulmonary hemodynamics to idiopathic PAH, thus making their classification of PH challenging. Such cases should prompt a referral to centers of expertise for appropriate management [82].

The benefit of PAH therapy in non-IPF fibrotic lung diseases remains unclear and is presently limited to retrospective studies [93]. Riociguat, a soluble guanylate cyclase stimulator, has demonstrated some efficacy in initial trials of patients with PAH (Group 1), PH associated with FLD (Group 3), or chronic thromboembolic pulmonary hypertension (Group 4) [89, 94–97]. However, larger well-designed clinical trials are needed before adaptation for widespread use [90].

### 6.3. Therapies for Pulmonary Arterial Hypertension

#### 6.3.1. Calcium Channel Blockers

The dihydropyridine calcium channel blockers such as nifedipine and amlodipine appear to be safe and well tolerated in patients with a positive pulmonary vasoreactive test and may confer a survival benefit in these individuals. However they may exert potentially negative inotropic effects with long-term consequences that remain unclear [98].

#### 6.3.2. Endothelin (ET-1) Receptor Antagonists (ERA)

Endothelin-1, a potent vasoconstrictor produced by vascular endothelial cells and cardiomyocytes, also mediates the regulation of several biological processes in other tissues outside the cardiovascular system [99–102]. The effects of ET-1 are mediated via two different receptor subtypes, ET_A_ and ET_B_. Endothelin (ET-1) receptor antagonists directly oppose its effects on cardiomyocyte contractility and the indirect effects on pulmonary vascular remodeling and vasoconstriction [103]. Bosentan, a nonselective receptor antagonist, was the first ERA to receive FDA approval for PAH in patients with WHO-FC III or IV. It has however been associated with sporadic increases in aminotransferases and anemia [104–107]. Ambrisentan, an ET_A_ selective antagonist, also improves exercise capacity with the added benefit of once daily dosing and a reduced tendency to cause aminotransferase abnormalities [108, 109]. Macitentan, the most recent oral ERA approved for use in these patients, was demonstrated to have a 45% reduction in morbidity and mortality and potential for use in patients with inoperable chronic thromboembolic pulmonary hypertension [110].

#### 6.3.3. Prostacyclin Analogs

Prostacyclin (also called prostaglandin I_2_ or PGI2), a molecule that mediates vasodilation, inhibits platelet aggregation and inflammation and vascular smooth muscle proliferation also has important direct cardiac effects [111, 112]. Synthetic PGI2 analogs such as epoprostenol (Flolan) improve right ventricular stroke work and have demonstrated survival, functional, and hemodynamic benefits in patients with PAH [113–116]. The significantly short half-life (3–5 min) and instability at room temperature presented practical challenges and more stable and convenient formulations (Veletri) have recently been made available with similar effects on pulmonary hemodynamics. Other PGI2 analogs such as treprostinil (which may be administered subcutaneously or intravenously or inhaled) and Iloprost (inhaled) may be used as alternative therapies [117–121]. Their direct effects on right ventricular function remain unclear and the initial improvement in exercise capacity observed with oral PGI2 analogs after 12 weeks has been reported to disappear after 1 year [122]. Furthermore, treatment with epoprostenol for 6 months has been reported to be associated with increased mortality, an effect that may be explained by the detrimental consequences of increasing myocardial oxygen consumption when contractility increases [112, 123, 124].

#### 6.3.4. Phosphodiesterase Type 5 (PDE5) Inhibitors

The formation of the intracellular messenger, cyclic guanosine monophosphate (cGMP), a potent smooth muscle relaxant and pulmonary vasodilator, is induced through activation of soluble guanylate cyclase (sGC) by nitric oxide (NO), a short-acting molecule produced by vascular endothelial cells [125]. The phosphodiesterase type 5 (PDE5) enzyme degrades cGMP; thus oral PDE5 inhibitors such as sildenafil and tadalafil result in significant vasodilatory and antiproliferative effects [126–128]. Sildenafil has been demonstrated in patients with idiopathic pulmonary fibrosis and right ventricular dysfunction to improve quality of life and preservation of exercise capacity [88].

#### 6.3.5. Soluble Guanylate Cyclase (sGC) Stimulators

A recent sGC stimulator, Riociguat, independently increases cGMP levels and improves WHO functional class, pulmonary vascular resistance, and serum markers of right ventricular stress [129].

#### 6.3.6. Rho-Kinase (ROCK) Inhibitors

These hold significant promise for treatment of RHF in severe PH and their acute administration results in modest pulmonary vasodilation [130, 131]. Their long-term effects on the right ventricular are unknown but a recent study of 74 patients who received fasudil, an intravenous rho-kinase inhibitor, demonstrated mortality benefits and reduced hospitalization and a favorable side effect profile [132]. The efficacy of statins and histone deacetylases in pulmonary hypertension has also been evaluated in multiple studies with limited success [133–136].

#### 6.3.7. Other Connective Tissue Disease Specific Therapies

Inhaled nitric oxide (iNO) has been studied to examine its effect on pulmonary vasoreactivity in patients with systemic sclerosis (SSc) who demonstrate pulmonary hypertension and right ventricular failure [137]. A study of 60 patients found no response to iNO in diffuse SSc. Though 40% of patients with vasoreactivity to iNO had pulmonary fibrosis, patients with no vasoreactivity more commonly exhibited fibrosis typical of diffuse scleroderma [137]. Decreased pulmonary pressures after administration of iNO were associated with subsequent improvement in right ventricular systolic function [137].

In patients with systemic lupus erythematosus-associated pulmonary arterial hypertension (SLE-PAH), intensive immunosuppressive therapy with intravenous cyclophosphamide and oral glucocorticoids has been demonstrated to decrease mPAP and improve hemodynamic parameters, six-minute-walk distance, and survival [138–141].

#### 6.3.8. Emerging Treatment Options

Oral prostanoids such as Beraprost (twice daily dosing) and treprostinil (thrice daily dosing) have been evaluated as monotherapy with mixed results but are currently under investigation in various trials for their utility as combination therapies [122, 142, 143]. Selexipag, an oral, nonprostanoid selective IP receptor agonist, demonstrated a 39% reduction in time to first morbidity or mortality over a 4-year period and is currently being evaluated for its safety profile [144, 145]. Vardenafil, an oral PDE5 inhibitor, has been demonstrated to improve pulmonary hemodynamics and exercise capacity at 12 weeks while reducing oxidative stress. It however remains under investigation for treatment efficacy in patients with PAH [146, 147]. Tyrosine kinase inhibitors such as imatinib have demonstrated treatment benefit in isolated cases, an effect that has not yet been replicated by several trials, some of which were discontinued due to severe side effects [148–155].

## 7. Effects of Antifibrotic and Anti-Inflammatory Agents on Heart Failure in Idiopathic Pulmonary Fibrosis

Two new agents have recently been approved for the treatment of patients with IPF. Pirfenidone is an oral antifibrotic agent with mechanisms of action that include the inhibition of key cytokines that mediate pathogenesis of inflammation and fibrosis [156]. Nintedanib is an oral intracellular inhibitor of tyrosine kinase that targets multiple growth factor receptors [157]. Both agents have been shown in multiple randomized controlled phase 3 trials to slow the rate of decline in lung function of patients with IPF [156, 157].

A multinational comprehensive evaluation of the long-term safety of pirfenidone in patients with idiopathic pulmonary fibrosis found no increased incidence in adverse cardiac events [158]. Interestingly, pirfenidone has been demonstrated in various animal models to attenuate myocardial fibrosis and left ventricular remodeling by inhibiting NLRP3-induced inflammation and subsequent fibrosis [159, 160], ultimately resulting in cardioprotective effects [161, 162]. However, these findings have not yet been demonstrated in human studies. Two large trials examining the efficacy and safety of nintedanib in patients with IPF did not demonstrate a significant increase in the incidence of cardiac adverse effects with the use of this medication [157].

## 8. Right Ventricle-Targeted Therapies

The initial cardiac hypertrophy, which occurs in response to the prolonged increase in pulmonary vascular pressure and altered hemodynamics, progressively becomes maladaptive and eventually results in decompensated ventricular function. As PH progresses, right ventricular dilation and fibrosis follow eventually resulting in functional right ventricular failure, the most common cause of death in patients with severe PH [24, 163, 164].

The persistently poor prognosis of patients with low right ventricular function despite therapies that effectively reduce the pulmonary vascular resistance highlights the crucial need for right ventricular-targeted therapies in these patients [165]. The underlying mechanisms of right ventricular failure are increasingly thought to differ from that of the left ventricle and this may explain the variation in results across experimental therapies targeting both ventricles [166] (Table 1).

### 8.1. Pharmacologic Agents

#### 8.1.1. *β*-Adrenoceptor (*β*-AR) Blockers

Though downregulated *β*-adrenergic receptors and increased sympathetic activity are typical features of pulmonary arterial hypertension, use of these medications may decrease heart rate and myocardial contractility and result in systemic vasodilation limiting their unrestricted utility in these patients [167, 168]. Patients with portopulmonary hypertension also demonstrate poor functional capacity and worse pulmonary hemodynamics with use of these medications [169]. Significant benefits such as reduction of myocardial oxygen consumption, restoration of effective Ca^2+^ transport, and prevention of arrhythmias may be achieved with the careful use of these medications [170, 171]. Carvedilol, a selective *β*1-AR blocker, improves right ventricular function and exercise tolerance and is described to exert cardioprotective effects [172–174]. Use of carvedilol has also been described in experimental models to improve biventricular fibrosis [175]. Bisoprolol has been shown in animal studies to improve right ventricular-arterial uncoupling and survival [176]. The therapeutic benefits of inhibition of G protein-coupled receptor kinase-2 (GRK) mediated uncoupling of the *β*-adrenergic receptor have also been described with the use of Gallein, a novel small molecule that targets the G*βγ* subunit of GRK2 [177, 178].

#### 8.1.2. Angiotensin-Converting Enzyme (ACE) Inhibitors

The effect of ACE inhibitors on pulmonary hemodynamics and right ventricular function has not been evaluated by large studies. Limited data from case series yield conflicting results [179, 180]. Experimental animal models of ramipril describe an improvement in right ventricular systolic function [181].

#### 8.1.3. Modulators of Metabolism

Progression of right ventricular failure is accompanied by downregulation of fatty acid oxidation, which may contribute to the mechanistic process [182, 183]. Metabolic modulators like ranolazine or trimetazidine have been demonstrated to mitigate the reduction in cardiac output with modest effects observed in right ventricular dysfunction [166, 183]. Use of etomoxir, an inhibitor of fatty acid oxidation, has been shown to have equivocal results in right ventricular failure [184].

#### 8.1.4. Antioxidants

Administration of protandim in experimental PH models has been shown to upregulate the expression of HO-1 (hemoxygenase-1), an isoenzyme that facilitates the production of antioxidant enzymes by promoting the expression of their genes [166, 185].

#### 8.1.5. 5-Hydroxytryptamine-2B (5-HT2B) Receptor Antagonists

Murine models of pulmonary hypertension have demonstrated a significant role for 5-hydroxytryptamine (serotonin) in the development and progression of ventricular hypertrophy [186–188]. Terguride, a 5-HT2A and 5-HT2B receptor antagonist, and SB204741 (a 5-HT2B receptor antagonist) have been demonstrated to inhibit right ventricular fibrosis by reducing collagen deposition [189].

### 8.2. Nonpharmacologic Modalities

The efficacy of exercise rehabilitation and respiratory training in patients with pulmonary hypertension and heart failure has been studied and shown to improve exercise capacity, improve quality of life, and correct endothelial dysfunction [190, 191].

Cardiac resynchronization in PAH patients with ventricular dyssynchrony may correct the difference in duration of right ventricular contraction when compared to the left ventricle with subsequent improvement in right ventricular systolic function and diastolic relaxation [192–194]. Atrial septostomy may also be beneficial in severely ill patients with significantly elevated pressures by reducing the right ventricular preload [195, 196]; therapy should however be individualized to each patient and limited to centers with expertise at performing this procedure [166]. Mechanical right ventricular support with extracorporeal membrane oxygenation (ECMO) and ventricular assist devices (VAD) may also be necessary for temporary circulatory support [197–199]. The CentriMag (a short-term continuous-flow pump) and PVAD (a long-term pneumatic pulsatile pump) are circulatory assist devices recently approved by the FDA for right ventricular support [200, 201]. The Impella RP approved for use in Europe is being evaluated for its safety and efficacy in the USA for support of cardiac function in patients with right ventricular failure [202, 203].

## 9. Transplant for Treatment of Fibrotic Lung Disease

Progression of advanced pulmonary fibrosis that remains refractory to medical management may eventually require single- or double-lung transplantation. A study of 821 recipients of lung transplant for pulmonary fibrosis showed significantly better early and late survival in recipients aged < 60 years with single-lung transplant than bilateral lung transplant. Patients with IPF tend to be >60 years and in studies focused on IPF patients, double-lung transplant may be associated with equivalent or better long-term outcomes and graft survival than single-lung transplant [204, 205]; however unilateral transplant is an acceptable alternative and may affect the allocation process [206]. The preoperative mean PAP (<40 mmHg) has been demonstrated by multivariate analyses to be an independent risk factor for operative mortality (OR = 9.7; *p* = 0.01) [206]; younger patients with significant pulmonary hypertension may benefit from receiving bilateral lung transplant [206]. Patients with severe PAH and right ventricular dysfunction may be considered for combined heart-lung transplantation [166].

## 10. Conclusion and Future Directions

The rising prevalence and mortality from fibrotic lung diseases create an urgent need for improved therapeutic strategies in the management of right ventricular failure and PH in patients with fibrotic lung disease, as there is a significant limitation of organs available for transplant. The poor resting gas exchange, low diffusing capacity of the lungs for carbon monoxide (DLCO), and cardiovascular mediated exercise limitation that characterize this unique group of patients contribute to the mechanisms driving progression of right ventricular dysfunction to failure. Individualized therapy should be instituted early and target the underlying lung disease as well as those specific mechanisms leading to right ventricular failure. As new treatment options emerge, clinical trials should focus on development of therapies with the most efficacy and improvement in quality of life while considering the effects on right ventricular function.

## Figures and Tables

**Table 1:** Effects of pharmacologic therapies in patients with right ventricular dysfunction^*∗*^.

Medication	Route of administration	Mechanism of action	Right ventricular effect	Common side effects
Therapies targeting pulmonary hypertension				
Nifedipine and amlodipine	Oral	Calcium channel blockers	Reduce afterload	Headache, dizziness, and extremity edema
Bosentan, ambrisentan, and macitentan	Oral	Endothelin receptor antagonists	Reduce afterload	Headache, dizziness, and arrhythmias
Epoprostenol	IV	Prostacyclin analog	Reduces afterload	Nausea, vomiting, dizziness, and arrhythmias
Iloprost	Inhaled	Prostacyclin analog	Reduces afterload	Nausea, vomiting, headache, and diarrhea
Treprostinil	SC/IV/inhaled	Prostacyclin analog	Reduces afterload	Nausea, headache, cough, and dizziness
Sildenafil and tadalafil	Oral	Phosphodiesterase type 5 inhibitors	Reduce afterload	Nausea, vomiting, headache, and tritanopia
Riociguat	Oral	Soluble guanylate cyclase stimulator	Reduces hypertrophy	Headache, dizziness, gastritis, hypotension, and diarrhea
Imatinib	Oral	Tyrosine kinase inhibitor	Improves function	Nausea, vomiting, edema, diarrhea, rash, and pancytopenia
Fasudil	IV	Rho-kinase inhibitor	Reduces hypertrophy	Nausea, renal dysfunction, fever, and thrombocytopenia
Nitric oxide	Inhaled	Pulmonary vasodilator	Improves function	Hypotension and methemoglobinemia

Therapies targeting the right ventricle (RV)				
Carvedilol and bisoprolol	Oral	*β*-adrenergic receptor blockers	Decrease myocardial fibrosis	Dizziness, fatigue, diarrhea, and hyperglycemia
Ranolazine and trimetazidine	Oral	Modulators of metabolism	Decrease remodeling	Nausea, headache, dizziness, constipation, edema, and dyspnea
Ramipril	Oral	ACE inhibitor	Decreases myocardial fibrosis	Nausea, vomiting, cough, headache, and dizziness
Protandim	Oral	Antioxidant	Decreases myocardial fibrosis	Nausea, vomiting, rash, headache, and diarrhea

Therapies targeting pulmonary fibrosis^*∗∗*^				
Pirfenidone	Oral	Antifibrotic agent	Decreases myocardial fibrosis	Nausea, vomiting, rash, headache, diarrhea, and dizziness
Nintedanib	Oral	Triple angiokinase inhibitor	Undetermined direct effect	Nausea, vomiting, headache, diarrhea, and anorexia

^*∗*^None of these medications have been specifically approved for Group 3 pulmonary hypertension as these patients may have pulmonary fibrosis and may not demonstrate vasoreactivity. ^*∗∗*^Idiopathic pulmonary fibrosis, IV: intravenous, SC: subcutaneous.

**Table 2:** Trials of pulmonary hypertension therapies in idiopathic pulmonary fibrosis.

Trial	Design	Medication/dose	Primary endpoint	Outcome
BUILD-1 (Bosentan Use in Interstitial Lung Disease)	Randomized, double-blind, placebo-controlled, multicenter study	Bosentan (oral) 62.5 mg b.i.d. × 4 wk., then 125 mg b.i.d. ≥ 12 mth.	6-minute-walk distance	Bosentan showed no superiority over placebo

STEP-IPF (Sildenafil Trial of Exercise Performance in Idiopathic Pulmonary Fibrosis)	Randomized, double-blind, placebo-controlled trial	Sildenafil (oral) 20 mg t.i.d.	Proportion of patients with ≥20% increase in 6-minute-walk distance	Sildenafil showed no superiority over placebo in primary outcome

BUILD-3 (Bosentan Use in Interstitial Lung Disease)	Prospective, randomized, double-blind, placebo-controlled, event-driven, parallel-group trial	Bosentan (oral) 62.5 mg b.i.d. × 4 wk., then 125 mg b.i.d.,	Time to IPF worsening or death	No significant difference between treatment groups

ARTEMIS-IPF (Randomized, Placebo-Controlled Study to Evaluate Safety and Effectiveness of Ambrisentan in IPF)	Randomized, double-blind, placebo-controlled, event-driven phase 3 trial	Ambrisentan (oral) 10 mg daily	Reduction in rate of IPF progression	Early study termination due to worsening of lung function decline and increased respiratory hospitalizations in ambrisentan group

MUSIC (Macitentan Use in an Idiopathic Pulmonary Fibrosis Clinical Study)	Prospective, randomized, double-blind, multicenter, placebo-controlled, parallel-group phase 2 trial	Macitentan (oral) 10 mg daily	Effect on forced vital capacity	No differences in pulmonary function tests or time to disease progression or death

BPHIT (Bosentan in Pulmonary Hypertension Associated with Fibrotic Idiopathic Interstitial Pneumonia)	Randomized, double-blind, placebo-controlled phase 4 study	Bosentan (oral) 62.5 mg b.i.d. × 4 wk., then 125 mg b.i.d.	≥20% decrease from baseline of pulmonary vascular resistance index over 16 weeks	No difference in primary outcome
